# Extra Supplement—The Nursing Mirror

**Published:** 1889-09-07

**Authors:** 


The Hospital,
SEPTEMBER 7, 1889.

### The Nursing Mirror.

Extra Supplement

All Contributions for this Supplement should be addressed to the Editor, THE HOSPITAL, 139-140, Salisbury Court, Fleet Street, London, E.C., and should have the word "Nursing" plainly written in left-hand top corner of the envelope

### En Passant.

LUTON COTTAGE HOSPITAL.—A new room is being built to Luton Cottage Hospital to permit of a second nurse being added to the present small staff. The room proposed was 10ft. by 12ft. and 8ft. high, with staircase from the corridor, and window in the gable end. A stove was suggested, but the committee thought it could be arranged to have an open fireplace with chimney leading to one of the existing flues. Here is a good opportunity to calculate cubic feet of air!

A WOMEN'S PARADE.—What will those women do next? They are not only forming themselves into friendly societies, but they are taking to collecting in the cause of charity, just as though they were men! Last month a parade and demonstration was held in Deptford by the "Duchess of Albany" Lodge U.O.T.A.S.P., the first of the kind organised by the women. It was very successful, and the collection was divided between the lodge funds and the Hospital of the Nursing Sisters of St. John the Divine, Lewisham. One feature of the procession was a dog who had assisted in many charitable purposes.

BIRMINGHAM HOSPITAL.—An interesting ceremony took place on August 23, when about twenty nurses belonging to this hospital, who had completed their three years' training, were presented with silver medals. Each medal bore the name of the recipient and the date of the completion of her training. Mr. Barrow (chairman) addressed the nurses shortly, and then presented the medals. Dr. Coghill, house-governor, moved a vote of thanks to the chairman, which was quietly and modestly seconded by one of the recipient nurses. We are sure many will regard these medals as precious souvenirs of a happy three years' work in Birmingham.

MISS WHATELY'S WORK.—Some months ago we chronicled with regret the death of Miss Whately, but we are glad to learn her work in Egypt yet continues. The latest addition to the mission is a medical branch, carried on by Dr. Azoury, from Lebanon, and some idea of the success of this institution may be obtained from the fact that during the first twelve months some 3,000 patients obtained relief or were cured. Numbers of "halt, maimed, and blind," with all sorts of sufferers, attend, and very many find relief, while all have the opportunity of hearing something of Scripture, which is daily read to them in the dispensary.

NEW YORK NURSING.—A most excellent school of training for nurses is connected with Bellevue Hospital, New York; the nurses' home being at 426, East Twenty-sixth-street. The training is modelled on the Nightingale School, and was started by Sister Helen, of King's College Hospital. Each year the number of applicants for admission is so large, the committee can choose those likely to make the best nurses; last year there were about 400 applications, and about fifty were accepted. There are no paying probationers. Four courses of lectures are delivered during the year on Obstetrics, Anatomy, Physiology, and Diseases; and at the close of the year a doctor always gives a valedictory address to those who are leaving the school for other fields of labour. The custom is one which might well be copied in England. We have constantly quoted from these addresses in our pages.

NURSES AT ROME.—There exists in Rome an institution of trained nurses known as St. Paul's House. The nurses are trained chiefly in America, and the superintendent is Miss A. S. Martin, who graduated at Bellevue Hospital in 1882. The nurses get plenty of work from English and American visitors, and a doctor who lately visited them says their untiring zeal and intelligent care produce admirable results.

AN ECCENTRIC SISTER.—Sir Andrew Lusk lately ordered Mary Anne Leine, 50 years of age, who was brought before him, to be taken to the workhouse. The prisoner, who was dressed in the garb of a sister of mercy, said she came from Abberfeld Convent, Limerick. The poor woman had been found outside the Mansion House Station, in Cannon-street, where her behaviour was such as to lead the police to think she was of unsound mind. After the alderman's decision, it was stated that the prisoner had been claimed by the authorities of Nazareth House, Hammersmith, in which institution she had resided.

MALE NURSES.—We have received a letter from a Scotch correspondent, who considers himself aggrieved because we "never mention" anything about male nurses. Now, we do occasionally mention them; but we seldom receive news from them, so our notices of their doings are naturally small. Male nurses are certainly a great advantage, especially in hospitals, where a delirious case is often in a ward where only one female nurse is on duty. We have seen one of the Hamilton nurses sitting watching over a Guy's patient in a male medical ward. Amongst the "upper ten" also, gentlemen who are used to valets have often a strong objection to a woman nurse, and here the male nurse can do good work. Of course, in asylums the male attendant reigns alone in the male wards, and there are few women who would wish to replace him. There is no jealousy or distrust between nurses of either sex, we hope; and we will always welcome any news regarding male nurses or their work.

EXAMINATION QUESTIONS.—We have received a number of answers to the last lot of examination questions, and we propose to publish some of these replies. The most notable point in such answers as we have received is the total absence of punctuation: a dash is made to do duty for comma, semicolon, or full stop quite indiscriminately. The facts given are generally correct, though we believe that to "keep patient very warm" in a case of scarlet fever would not meet with the approval of all medicos; the cold treatment is coming into favour in scarlet-fever cases. Again, the reason for keeping a patient in bed for some time during convalescence after rheumatic fever is chiefly based on the fear that the heart may be affected, and sudden movement may be fatal: most correspondents say extra care in rheumatic fever is required, because of susceptibility to cold; but, certainly, the nursing of a case of rheumatic fever where no watch is kept for symptoms of heart trouble, would not be perfect nursing. The preparations for a capital operation in a private house include a carefully arranged table in a good light; this is a point our correspondents have missed, giving all their attention to details about basins, pins, etc. One nurse never even mentions any disinfectant. We are sure carefully written replies to these specimen examination questions would help many nurses to discover and remember nursing points likely to be passed over or forgotten.

### Physiology for Nurses.

### NO. VIII.—JOINTS AND MUSCLES.

BEFORE we can get any further, we must settle exactly what a joint is, and what its use is. A joint is at once the union and the division of two bones. You can only have a joint where two bones meet, as at the knee, for instance, where the thigh-bone joins the two bones of the leg. But in addition to joining the bones, a joint also helps them to move, which sounds like a contradiction, though it is not really so; for were the two bones joined so that they could not move, as happens when a person has a stiff knee which cannot be bent, then there is no longer a joint. So please try and remember that a joint both joins bones and enables them to move on each other. In the human body you will find three different kinds of joints: (1) the ball and socket joint, as, for instance, where the upper arm fits into the shoulder, and the upper part of the leg fits into the thigh: and here you have the end of the bone shaped round almost like a ball, and a deep cup into which its fits, and the ball can move round and round, and backwards and forwards, and up and down in this cup; in fact, this is the kind of joint which gives us most variety of movement. The next kind is called the (2) hinge joint, and it only allows the bones to work backwards and forwards, as a door moves; you get this kind of joint at the elbow and knee. But you will tell me we can move the lower part of the arm round just as we can the upper. So you can, but it is the ball and socket joint at the shoulder that moves the whole arm, not the elbow joint, and if anything happened to stiffen the ball and socket joint at the shoulder, the lower part of the arm would not be able to move round. You can settle this matter for yourselves, and make quite sure that it is so by grasping the upper part of the arm whilst you turn round the lower part, and you will find the whole arm is moving. The joints which move the hands and enable you to turn them round are an example of the third kind of joint—that is, the (3) sliding joint: the bones slip about on one another. The bones of the spine act in just the same way, and are, perhaps, a better example of this kind of joint; as the hand is able to move in various ways and has a wonderful variety of movement, and hence the delicacy of touch that you find in the human hand. How is it these joints work so easily and never give us any pain? In a fresh joint got at the butcher's you can see how the joints look in life. In this ball and socket joint, the round end of the bone, as well as the cup, are covered or lined with a beautifully smooth substance which is kept moist and smooth with what is called joint oil, a supply of which is found in a sort of bag just above and outside the joint, and a very small quantity is poured out as it is wanted. The cartilage, or gristle, as it is called, has no nerves in it, and therefore has no feeling; if it had, we should have pain when we moved. The bones are kept in place at the joints by very strong bands or ligaments; at the hinge joints a number of these bands are fastened above and below, but in the ball and socket joint these ligaments also surround the joint, and form a sort of cap, inside which the joint can freely move. In disease, when this smooth gristle or cartilage gets worn away, and the ends of bone rub together as they do in the bones of a skeleton, the pain is very great, because the bones have nerves, though the gristle which forms the buffer has none. A bone which has lost its gristle is like a decayed tooth, when the tooth has decayed down to the nerve. In a healthy tooth, the nerve is well covered up, and gives you no pain, but it is there all the same; and in a healthy bone the nerves are there, though they do not let you know it, and only make their presence felt when the cartilage is worn away. And this is the reason why hip disease is so extremely painful. Either the cartilage is altogether worn away, or the joint oil used up, so that the joint moves with difficulty, like the rusty hinge of a door; and there is great stiffness in it, for, naturally, if it is painful to move any particular limb or joint the owner will not move it more than can be helped. On the other hand, sometimes you get stiffness alone, without pain, and this equally shows that there is disease. You find hip disease chiefly amongst children—they are restless little creatures, never still for long together, and therefore much more likely than grown-up people to get some slight blow or other injury which is soon forgotten at the time, but may have more serious consequences behind. The hip joint, because it is capable of so much movement, does the most work and is liable to the most injury, and is the most often the seat of disease. To begin with, you must clearly know the part of the thigh-bone of which we are talking. It is not that ridge which is usually called the hip-bone: it is this other part, three or four inches lower down. It is well hidden from sight and touch, and securely surrounded by thick coverings of soft substances, such as fat, and sinew, and flesh, and skin. But though the hip joint itself is so well out of sight, when disease has got hold of it it gives very decided signs of its existence, but for a watchful person it is easy to recognise the warnings that disease is coming. Sometimes there is severe pain, however, at a very early stage which cannot be overlooked, and proper medical advice is sought. Children usually have hip disease between the ages of seven and thirteen, and it is more common among boys than girls because their amusements are usually more boisterous than those of girls. It usually attacks boys of weak constitutions who wish to take their share in the rough games of their companions, but cannot because they get tired sooner, and at the end of the day they seem little inclined for exertion, and seem only too glad to go to bed, a wish that is not usual with healthy boys. At night the boy may complain of a slight pain in the knee. This way that pain has of not always being in the part where the disease is, is very common, and is very misleading and unfortunate. Doctors recognise it, and even give it a special name, "referred pain," and would know what to look for. Why there should be pain in the knee when the hip is diseased you will understand better later on, when we speak of nerves, so just for the present you must take my word for it that it is so. After a night's rest this pain will be better, but will return again towards evening. Also on getting up in the morning there will probably be slight stiffness of the thigh, and the child will move about cautiously just at first, though as the day goes on the stiffness will disappear. This may go on for some months, but gradually, a careful observer will notice that the child gets into the habit, when standing still, of resting all his weight on one leg, which of course is the healthy one, and it has the extra work to do so as to save the diseased leg. This way of standing is very characteristic of hip disease, and will at once tell an experienced person what is the matter. If you tell such a boy to stand upright before you, you will notice that he plants the foot of the sound leg firmly on the ground, his body inclines slightly to that side, the diseased leg is a little bit bent at the hip and knee, the foot is placed a little forward, the toes rest lightly on the ground, only enough to just ease the painful joint of the weight of the leg, and he will, whenever possible, rest the hand of the good side on anything that is near, and prop himself up. Together with the appearance of this characteristic way of standing, the child will often limp a little towards evening, but not enough to attract attention, as a rule, or make the parents think there is anything wrong. Also at this time, which you understand is a step further on than when the pain was in the knee, the child will complain of pain in the hip itself. If all these warnings are overlooked, the next step is the appearance of an abscess round the joint; at first it is merely a pale swelling without any redness or inflammation. The appearance of the swelling always shows that the joint is destroyed, and crippling is almost inevitable. Those caps of smooth oily cartilage have melted away and gone, and the bare rough bones are now rubbing against each other, and every movement gives the unhappy child great pain. Nature renews most parts of our bodies, but she never gives the joints fresh cushions of cartilage. All she will do is to add more bony matter, and make a stiff joint by making the two bones into one, and now that the hips are fixed the lower part of the back has to do the work of the hip, and so you get the peculiar movement you may all have noticed in people. Nature's way in hip disease is very cruel if she is left to herself, for she not only stiffens the hip in its socket, but, unless art interferes, she will stiffen the leg in a position which makes walking very difficult indeed, for she binds the thigh upwards and inwards so that the leg would be fixed as if it was always crossed over the other. In these cases the child has to be fastened firmly into a splint, and kept always at rest on his back, so that since we cannot now help the hip joint from growing stiff we can, at least, make it grow stiff in a position that shall be the least possible inconvenience to the child.

### Nurses on Their Travels.

### ACROSS CANADA.

To begin with, the voyage from Liverpool to Quebec was anything but pleasant, and I promptly made up my mind never to become a nurse-stewardess. Montreal has a fine hospital "Hotel Dieu," the exterior only of which I had time to see. Toronto also has a good hospital, with an annex for inebriates. We had a splendid two days' run over Lake Superior; beautifully cool on the lake, though it was 90deg. in the shade on shore. A wilderness of rock and burnt poles between Port Arthur and Winnipeg, then some good land, then a boundless prairie beyond. Winnipeg is about 10 years old, and has 20,000 inhabitants. It has a nioe hospital with 50 inmates whilst I was there. There is a night ambulance, a capital arrangement. The railway journey across the prarie was long, and hot, and dirty, and tiring. I began to think it the most awful example of travelling I had ever come across, even worse than sea-sickness. Right through from Montreal to Vancouver takes six days' continuous railway journey; but we stopped at Banff springs and elsewhere. Of course, the scenery in some parts, about Banff, for instance, is lovely. At Mitford I sat up half the night on the platform with a red lamp to stop the express. No one else seemed inclined to do it, for everyone is fearfully independent here, and we were bound to get on somehow. At another station our luggage was dropped on to the prarie instead of the platform, and took half an hour to find in the rain and darkness. The line from Banff to Vancouver is a caution. At one place the grade is 5ft. in 100, and every mile or so there is a safety switch on which to slip the train in case she runs away, which has happened once or twice to freight trains.

At last, however, we safely reached Vancouver, where comfort and an hospitable welcome awaited us. The climate is splendid (so far, we have only been here in July as yet). Night temperature does not exceed 60deg. Fahr., and during day in shade about 80deg. Fahr. Vancouver is three years old; three years ago it was burnt down all but a few cottages. Now there are substantial stone buildings, and snug wooden houses for private folk. The City Hospital is an airy wooden building in the south of the town; it is nursed by two Miss Crickmays, the eldest of whom acts as matron. I believe she trained at Guy's. There is an assistant nurse, a night nurse, a porter, and five visiting surgeons. The ground floor ward has seventeen beds, and there were twelve occupied when I was there; one with a bad case of Bright's disease, one with erysipelas, and one with an ill-defined form of fever. The first floor is subdivided into two wards. Two rooms for special cases adjoin the nurses' bedrooms, with a glass panel between, so that the patients can be watched. The hospital is maintained by the city funds. St. Luke's Home takes nearly all female patients in Vancouver. I told you how excellently it was managed by Sister Frances. Any case of sickness is taken (not notoriously infectious), including typhoid. Those patients who can pay do so, others are kept free of expense. There are three sisters, and they do a great deal of private and district nursing as well as attending to the home. No good work seems to come amiss to them. Smallpox cases are isolated in a small quarantine home on an island in the harbour, close to the town. Such are the nursing and hospital arrangements of Vancouver. Personal experience I will relate to you later on.

### The General Hospital, Birmingham.

### SPEECH OF THE CHAIRMAN BEFORE DISTRIBUTING MEDALS TO NURSES, AUG. 23RD, 1889.

As chairman of the hospital it is my privilege to present the first batch of medals to the nurses of the institution, but while I feel it a great honour to distribute them, I admit I also feel an amount of responsibility lest I should let an opportunity pass a more experienced chairman would embrace, to offer some useful advice to the nurses assembled. Let me say at once I regard the vocation of a hospital nurse as a noble one. Could, for instance, any lives be more beautiful, or more honourable, than those of Florence Nightingale, and, nearer home, of Sister Dora, and noble women of their stamp? Of course I am well aware all nurses must not aspire to the eminence the two ladies I have named attained, but all of you may do good work, work of which any woman may be proud, and all work, the contemplation of which will prove a blessing to you in your declining years. Without wishing for a moment to lecture you, may I remind you that, in addition to your ordinary calling, that of ministering to the physical needs of the patients entrusted to your care, you may, and I am thankful to know many of you do, render them many kindly offices. You may listen to and often lessen their troubles, you may take an interest in their home belongings and volunteer good advice at a time when patients are more than ordinarily susceptible. Has it occurred to you, you receive your medals to-day under singularly happy circumstances? You receive them in this board-room, hallowed by the noblest traditions, in a room where perhaps more has been planned for the alleviation of human suffering than in any other room within a hundred miles or more of it. You see before you the magnificent portait, by Sir Joshua Reynolds, of Dr. Ash, the founder of the hospital, and you are surrounded by the busts and portraits of many of the distinguished men, both medical and lay, who devoted themselves to make this hospital useful and famous, and, by special request of the committee, you receive your decorations at the hand of the chairman, a man inferior to his predecessors, but one who loves the hospital, and who gratefully acknowledges that he has spent during the last twenty years many happy and profitable hours within its walls, and one who takes an interest in you nurses and in everyone connected with the institution. One word of advice, in conclusion, be loyal yourselves, and encourage the junior nurses to be loyal to your superior officers and to the committee, and I ask each of you, individually, to resolve that, so far as in you lies, you will do your utmost to maintain and extend the usefulness and renown of our ancient institution.

### Everybody's Opinion.

*Correspondence on all subjects is invited, but we cannot in any way be responsible for the opinions expressed by our correspondents. No communications can be entertained if the name and address of the correspondent is not given, or unless one side of the paper only be written on.*]

### THE PENSION FUND.

Nurse E. Wilson, writes: It is with unspeakable pleasure that I have read from time to time the interesting accounts of the National Pension Fund, and, as "One of the First Thousand," I wish to offer my most hearty thanks to all those kind gentlemen who have taken such an interest in us. How happy they must feel in doing so much good, for their work is a labour of Christian love. Their aim is to keep the instruments bright for daily use, for we must feel glad and happy in the thought that our future is cared for, and in ministering to our need are they not ministering also to the thousands of sick people on whom we wait? I should like to thank everybody who has rendered and who is now rendering such liberal assistance to our National Pension Fund.

### MALE NURSES.

Trained Male Nurse writes: I have been a constant and careful reader of your paper since its commencement, and have obtained many hints that have assisted me in my work. I am a nurse, but a male one, consequently I have an amount of embarrassment in addressing you, but what I wish to ask is why you never mention anything about the hundreds of male nurses and attendants that are in constant work in London and the provinces? It is a grievance among us that we are never thought of in the many discussions that are taking place to benefit the nursing world. Our work is of a kind that will never compete with women nursing, yet we have our troubles like the rest, which we should much like to air. Will you raise your powerful voice on our behalf? I am writing this on my own and five other attendants' behalf.

### Presentation.

Mr. T. A. Collinson, house surgeon at Durham County Hospital, has been presented by the nurses with a handsome set of brass table ornaments on the occasion of his resignation.

### Notice.

Further particulars regarding the Doll Show will appear next week, till which date the names of competitors will still be received.

### TO NURSES.

To you will be given the privilege of forming those lasting friendships which so often are forged in those dark and trying hours when the golden bowl is being broken, and the silver cord is being loosened. There is engraven on my memory the kind sad face of a nurse who came to me in the stillness of one of the saddest nights of my life, and quietly said: "Doctor, there is a change in the patient." There was no hurry or terror in her manner, and when I reached the bedside of my loved one and saw at a glance that the death-blow had been struck, as I turned to the nurse and saw that she had realised it all, and in her face read that deep, tender sympathy which one human heart can give another, I realised that it was a nurse's privilege to extend human sympathy to a soul struggling under the first shock of a great sorrow.—Prof. Alfred Loomis, M.D.

### Appointments.

[It is requested that successful candidates will send a copy of their applications and testimonials, with date of election, to The Editor, The Lodge, Porchester-square, W.]

Norfolk and Norwich Hospital.—The committee have appointed R. Wentworth White, Esq., M.R.C.S., L.D.S., to the newly-created post of honorary dental surgeon to the Norfolk and Norwich Hospital.

Kensington Infirmary.—The Guardians have appointed as matron of their infirmary Miss Frances Marian Hughes, who was trained under the auspices of the Metropolitan and National Nursing Association at the Royal Free Hospital from 1877 to 1880, was afterwards for two years lady superintendent of the Nurses' Institute at Canterbury, and has for the past three and a half years held the post of matron of the infirmary of the parish of St. George-in-the-East.

### Vacancies.

The following vacancies are announced:

**Matron.**

Salford Union Infirmary, £80.

**Nurses.**

Blackheath Nurses' Institute (several).

Bradford Infectious Hospital, £23.

Carlisle Infirmary (night), £24.

Cheltenham Institution.

Corporation Hospital, Bootle, £25.

Guisborough Hospital (head), £30.

Heart Hospital, Soho-square.

Hospital for Epilepsy, Regent's Park.

Hull Nurses' Home (several), £25.

Isle of Wight Nursing Institution (two).

Jersey Nurses' Institute.

Kent Nursing Institution.

Llandudno Cottage Hospital.

Lock Hospital, Harrow-road.

Morley Convalescent Home.

North Surrey District School (night), £25.

Nurses' Home, Newcastle.

Nursing Establishment, Bowdon.

Regent-street, Nottingham.

South-Eastern Fever Hospital, £30.

Southport Infirmary, £25.

Southport Nurses' Home (several).

Stepney Hospital for Children, £25.

Suffolk General Hospital, £25.

Sydney Nurses' Home (twelve).

Tunbridge Wells Hospital, £25.

Victoria Home, Bournemouth, £25.

West Bromwich Hospital, £22.

Westminster Ophthalmic Hospital, £14.

**Probationers.**

Great Yarmouth Hospital.

Horton Infirmary, Banbury.

Stroud Hospital, Gloucestershire, £15.

Wirral Children's Hospital.

### Notes and Queries.

To Correspondents.—1. Questions may be written on post-cards. 2. Advertisements in disguise are inadmissible. 3. In answering a query please quote the number. 4. If a private answer is desired, a stamped addressed envelope must be forwarded.

Notice.—We must really ask our correspondents to be more particular in enclosing name and address. Constantly we receive queries or letters with no name attached.

**Queries.**

(77) *Massage.*—Does either Dr. von Mosenziel or Dr. Metzger train pupils in massage as performed by them? *Aberdeen.*

(78) *W. M.* writes: What are the best agencies at which to apply for post of companion to invalid for the winter?

(79) *E. W.* asks for any information about nursing homes in India, and where to apply to for particulars concerning them.

(80) *K. B.* enquires (1) what steps should be taken to become a member of the Red Cross Society. (2) What hospitals in Germany or in Austria would be likely to admit an English trained nurse who can speak German and French?

**Answers.**

(71) I have found Cullingworth's "Manual for Monthly Nurses" very useful. Published by Churchill, New Burlington-street. Price 1s. 6d. *Doris.*

(76) *Home Wanted.*—Apply to the British Home for Incurables, 380, Clapham-road, or the Royal Hospital for Incurables, West Hill, Putney.

*Certificated Nurse.*—The library at 15, Buckingham-street is only open to members of the institute.

### NO MATTER WHO.

No matter who, so he thy service need,
No matter what the suppliant's claim may be
Thou dost not ask his country or his creed,
To know he suffers is enough for thee.

